# Ferroptosis-Related Gene Signature Accurately Predicts Survival Outcomes in Patients With Clear-Cell Renal Cell Carcinoma

**DOI:** 10.3389/fonc.2021.649347

**Published:** 2021-04-30

**Authors:** Kaili Chang, Chong Yuan, Xueguang Liu

**Affiliations:** Department of Pathology, School of Basic Medical Sciences, Fudan University, Shanghai, China

**Keywords:** ferroptosis, clear-cell renal cell carcinoma (ccRCC), prognostic, signature, nomograph

## Abstract

As a type of regulated cell death induced by Ras selective lethal (RSL) compounds such as erasti, ferroptosis is characterized by iron-dependent lipid peroxide accumulation to lethal levels. At present, little is known about the role of ferroptosis-related genes in clear-cell renal cell carcinoma (ccRCC). In the present study, the expression data of ferroptosis-related genes in ccRCC were obtained from the Cancer Genome Atlas (TCGA), and COX regression analysis was performed to construct a risk model of ferroptosis prognostic signature. The GEO database was used to verify the accuracy of the model. The following findings were made: the results reveal that the prognostic signature constructed by 11 ferroptosis genes (CARS, CD44, DPP4, GCLC, HMGCR, HSPB1, NCOA4, SAT1, PHKG2, GOT1, HMOX1) was significantly related to the overall survival (OS) of ccRCC patients based on the lowest Akaike information criterion (AIC); multivariate analysis indicates that ferroptosis-related gene prognostic signature was an independent prognostic factor in ccRCC patients; the calibration curve and c-index value (0.77) demonstrate that the nomogram with the signature could predict the survival of ccRCC patients; and enrichment analysis shows that the high-risk group were enriched in humoral immunity and receptor interaction pathways. The aforementioned findings indicate that the ferroptosis-related gene signature can accurately predict the prognosis of ccRCC patients and provide valuable insights for individualized treatment.

## Introduction

Renal cell carcinoma (RCC), also known as renal cell adenocarcinoma, is one of the most common malignant tumors, with an incidence of about 3% of all adult malignancies (1) and more than 90% of primary renal tumors and pelvic cancer (2). clear-cell renal cell carcinoma (ccRCC) is the most common subtype of renal cell carcinoma, accounting for 70–80% of renal cell carcinoma and has the highest mortality rate (3). In recent years, with the improvements of clinical diagnostic technology and molecular targeted therapy, great progress has been made in the diagnosis and treatment of ccRCC, including the emergence of new therapies such as immunotherapy based on anti-PD-1/PD-L1 inhibitors (2, 4, 5). However, more than 100,000 renal cell carcinoma patients die each year due to cancer progression (6). Therefore, identification of biomarkers and potential targeted drugs for individualized therapy is the key to determine the progress and prognosis of ccRCC (7).

Ferroptosis is an iron-dependent form of regulated cell death, which is driven by the lethal accumulation of lipid peroxidation (8). It is a novel death phenotype distinct from apoptosis, various forms of necrosis or autophagy (9). Compared with normal non-cancer cells, cancer cells have an increased demand for iron, which makes ferroptosis inducers potential for cancer treatment (10, 11). For example, CD8+ T cells activated by immunotherapy can enhance the iron sag of tumor cells, which is helpful to the anti-tumor effect of immunotherapy (12). Drug-tolerant persister cancer cells are vulnerable to GPX4 inhibition. Loss of GPX4 function results in selective persister cell ferroptotic death *in vitro* and prevents tumor relapse in mice (13). Some studies have shown that ferroptosis plays an important role in ccRCC. In renal cell carcinoma, glutathione is essential to prevent ferroptosis caused by impaired clear cell lipid metabolism (14). Inhibition of β-oxidation and reduced fatty acid metabolism makes renal cancer cells highly dependent on GSH/GPX pathway to prevent lipid peroxidation and cell ferroptotic death (15). Hence induction of ferroptosis has become a promising treatment for ccRCC.

Recently, Wu et al. used a lasso regression model to identify five out of 36 ferroptosis-related genes as the most powerful prognostic markers in ccRCC patients (16). However, considering the limitation of treatment strategy of renal clear cell carcinoma, new prognostic models need to be developed. In order to predict the survival outcome of ccRCC patients more accurately, we screened and identified 11 out of 60 ferroptosis-related genes to construct Cox proportional hazards model. We also produced a nomogram, hoping to provide new insights for the personalized treatment of ccRCC patients.

## Materials and Methods

### Data Collection

The RNA sequencing (RNA-seq) data and corresponding clinical data of 537 ccRCC patients were downloaded from the official website of TCGA (https://portal.gdc.cancer.gov/). Exclude samples with a patient survival time of 0 (n = 4). In addition, samples with incomplete clinical information will be deleted in the correlation study of clinicopathological characteristics. Thirty-nine ccRCC patients’ data obtained from GEO database (GSE29609) for external verification. Then, 60 ferroptosis-related genes were retrieved from the previous literature (17). The data from TCGA are public, so this study does not require the approval of the local ethics committee.

### Construction and Evaluation of Prediction Signature

Univariate Cox regression analysis was used to determine ferroptosis-related genes whose expression levels were significantly correlated with overall survival (OS) in ccRCC patients (*P <*0.05). Subsequently, multivariate Cox regression analysis was performed on the candidate ferroptosis-related genes, and the prognostic model was determined based on the lowest AIC value. The risk score was calculated according to the expression level of each gene and its corresponding regression coefficient (18). The formula is established as follows: $Risk\;Score=\Sigma_{i=1}^{n}Coef(i)\times x(i),where\;Coef\;(i)\;and\text{​ }x(i)$ represent the regression coefficient and the expression value of each prognosis related ferroptosis gene, respectively.

According to the median value of the risk score, patients were divided into high-risk groups and low-risk groups. The Kaplan–Meier survival curve compared the overall survival rate of patients in the high and low-risk groups. Principal Component Analysis (PCA) to explore the distribution of different populations. Time-dependent ROC curve analysis to evaluate the predictive power of gene signature and various clinicopathological features.

### Correlation Analysis Between Prognosis Characteristics of Ferroptosis-Related Genes and Other Clinicopathological Parameters

Stratified survival analysis was used to test whether the prognostic characteristics are highly accurate in different clinicopathological characteristics groups. Wilcoxon test was used to analyze the correlation between prognosis and clinicopathological features of ferroptosis-related genes in ccRCC patients. In addition, univariate and multivariate Cox regression analysis determine whether the prognostic signature related to ferroptosis was an independent predictor independent of other clinicopathological characteristics. N stage did not participate in the analysis, because half of the patient data is missing.

### Establishment and Validation of Nomogram

The nomogram was constructed from clinicopathological characteristics (age, gender, grade, stage, TMN stage) and risk scores based on prognostic signature, and predicts OS of ccRCC patients at 1, 3 and 5 years. C-index and the calibration curve were used to evaluate the predictive ability of the nomogram.

### Verification of Express Level and Prognostic Significance

Wilcoxon test was performed to compare the transcription level expression of tumor tissues and non-tumor tissues adjacent to cancer. The Human Protein Atlas (HPA) online database (https://www.proteinatlas.org/) was used to detect the expression of related genes at the translation level (19). Kaplan–Meier survival curve was performed to verify the prognostic value of 11 ferroptosis-related genes in ccRCC.

### Functional Enrichment Analysis and Gene Set Variation Analysis (GSVA)

According to the risk score, ccRCC patients were divided into high and low risk groups, |LogFC| >1, FDR-pvalue <0.05 were used as the cut-off criteria to obtain differentially expressed genes (DEGs). The biological functions of these differentially expressed DEGs were comprehensively detected by gene ontology (GO) enrichment and Kyoto genome Encyclopedia (KEGG) analysis methods to clarify the biological functions and pathways related to risk score. Gene Set Variation Analysis (GSVA) was used in association of prognostic gene signature and cancer hallmarks (20). The hallmark gene set was used to obtain the GSVA score of each gene set of each sample of ccRCC. Limma package (21) was used for difference analysis, and adj. P. Val <0.05 and the t value >2 was considered as significant.

### Statistical Analysis

In this study, Strawberry Perl for windows (Version5.18.2) was used for data processing, and R (3.6.2) was performed for data analysis. *P <*0.05 was considered statistically significant.

## Results

### Construction and Validation of a Ferroptosis-Related Genes Prognostic Signature in ccRCC Patients

**Figure 1:**
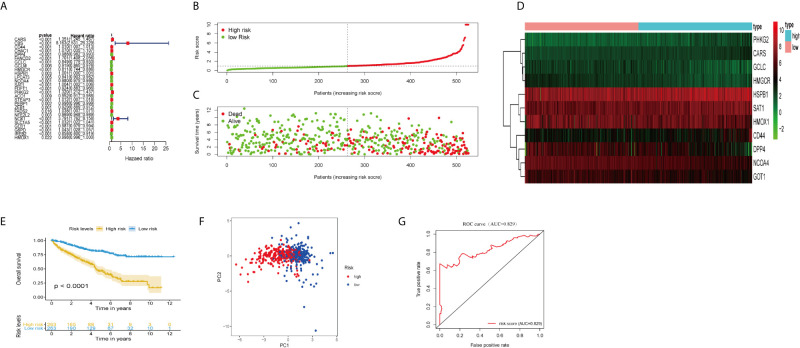
Construction and validation of the ferroptosis-related genes prognostic signature in ccRCC patients. **(A)** Univariate Cox regression analysis showed that 27 ferroptosis-related genes were related to the overall survival rate (OS) of ccRCC patients. **(B)** Distribution of risk scores of high- and low-risk BCLA patients based on the ferroptosis-related genes prognostic signature. **(C)** The scatter plot showed the correlation between the survival status of high- and low-risk ccRCC patients. **(D)** The heat map showed the differences in the expression of 11 ferroptosis-related genes between high and low risk patients. **(E)** Kaplan–Meier survival curve showed that the survival time of the high-risk group was significantly shorter than that of the low-risk score group. **(F)** PCA showed that 11 ferroptosis-related genes were distributed differently between high-risk groups and low-risk groups. **(G)** ROC curve analysis of risk score as an independent prognostic factor.

We extracted RNASeq data of ferroptosis-related genes from tissue samples of ccRCC patients in TCGA database. Univariate Cox regression analysis showed that 27 out of 60 ferroptosis-related genes were significantly associated with overall survival (OS) of ccRCC (*P <*0.05; **Figure 1A**). Multivariate Cox regression analysis indicated that 11 out of 27 ferroptosis-related genes were the best candidates to construct the prognosis signature based on the lowest Akaike information criterion (AIC).

Calculate the risk score of ccRCC patients according to the formula: risk score = (0.217 × CARS expression level) + (0.0098 × CD44 expression level) + (−0.0053 × DPP4 expression level) + (−0.307 × USP30-AS1 expression level) + (0.103 × GCLC expression level) + (−0.151 × HMGCR expression level) + (−0.0006 × HSPB1 expression level) + (−0.0094 × NCOA4 expression level) + (−0.0024 × SAT1 expression level) + (0.1523 × PHKG2 expression level) + (−0.007 × GOT1 expression level) + (−0.0024 × HMOX1 expression level). The median risk score (= 0.961) was used as the cut-off point to divide ccRCC patients into high- and low-risk groups (**Figure 1E**). The scatter plot showed that the survival rate of high-risk ccRCC patients was significantly lower than that of the low-risk group (**Figure 1F**). We also drew heat maps to show the expression levels of ferroptosis genes in high-risk and low-risk patients (**Figure 1G**). Kaplan–Meier curve displayed that OS of high-risk group was significantly worse than that of low-risk group (*P <*0.001; **Figure 1B**). PCA showed that patients in different risk groups had different distribution patterns (**Figure 1C**). The area under the time-dependent ROC curve (AUC) was 0.829, manifesting its excellent predictive performance (**Figure 1D**). We further verify our conclusion through GSE29609 dataset. The survival curve confirmed that high-risk patients had worse prognosis (**Figure S1A**; P <0.05). The AUC was 0.730, which indicated that the prognosis signature of ferroptosis-related genes could accurately predict the survival outcome of patients (**Figure S1B**).

### Correlation Between the Prognosis Signature of Ferroptosis-Related Genes and Clinicopathological Features

**Figure 2:**
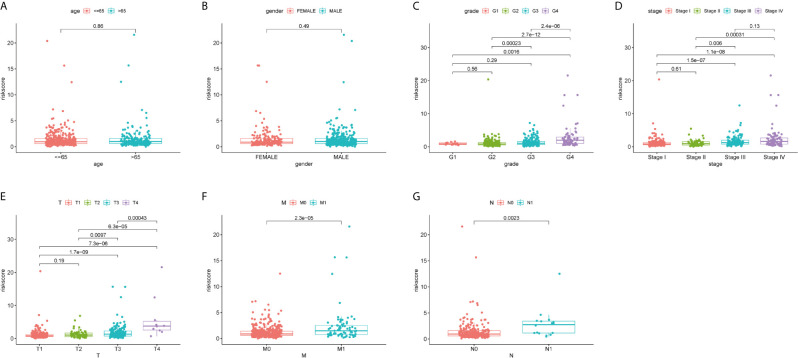
Correlation analyses of the ferroptosis-related genes prognostic signature with various clinicopathological characteristics of the ccRCC patients. According to **(A)** Age (≤65 y *vs.* >65 y); **(B)** Gender (male *vs.* female); **(C)** Grade (G 1/2; G3/4); **(D)** Stage (stage I/II; stage III/IV); **(E)** T stage (T1/2; T3/4); **(F)** M stage (M0 *vs.* M1); **(G)** N stage (N0 *vs.* N1) for analysis and comparison.

We explored the relationship between the risk scores and clinicopathological characteristics, and whether the risk scores have accurately predictive power for clinicopathological characteristics or not. As shown in **Figures 2A, B**, there was no difference in risk scores between gender and age (age ≤65 and age >65). However, as to grade, stage, and TMN stage, risk scores were significantly different in different groups and showed similar tendency (**Figures 2C–G**). With increase of grading or staging, the risk score was also rising.

Then a stratified analysis was carried out, according to age (≤65 and >65), gender (female and male), grade (grades 1–2 and grades 2–3), stage (stages I–II and stages III–IV), T stage (T1–2 and T3–4) and M stage (M0 and M1). N stage was eliminated due to missing data of some patients. As predicted, in all different clinical groups, the risk score based on the predictive signature of ferroptosis-related genes can accurately distinguish the prognosis of patients (**Figures S2A–L**; all *P* value <0.01). These results indicate that the risk signature of ferroptosis-related genes was related to the clinicopathological characteristics of ccRCC patients and could accurately predict the prognosis of ccRCC patients.

### Evaluation of Independent Prognostic Factors

**Figure 3:**
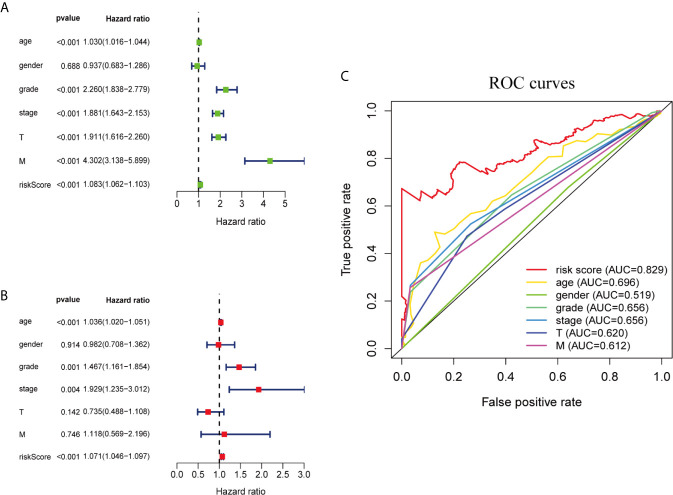
Ferroptosis-related genes prognostic signatures and other clinicopathological variables were used to evaluate the prognostic accuracy of ccRCC patients. **(A)** Univariate Cox regression analysis showed the correlation between overall survival rate and various clinicopathological parameters (Age, Gender, Grade, Stage, T and M stage). **(B)** Multivariate Cox regression analysis showed that age, grade, stage and risk score were independent prognostic indicators for overall survival of ccRCC patients. **(C)** ROC curve analysis showed the prognostic accuracy of risk score and clinicopathological parameters.

We performed univariate and multivariate Cox regression analysis to determine whether ferroptosis-related prognostic signature is independent prognostic factors in ccRCC patients or not. Univariate analysis showed that except gender, other clinicopathological features and prognostic signature of ferroptosis-related genes were significantly correlated with OS (*P <*0.001; **Figure 3A**). Multivariate analysis indicated that age (*P <*0.001), grade (*P* = 0.001), stage (*P* = 0.004) and the risk score of ferroptosis-related genes (*P <*0.001) were independent prognostic factors for ccRCC patients (**Figure 3B**). Multi-factor ROC curve demonstrated that the risk score had better predictive performance than the other clinicopathological characteristics, and AUC was greater than age (0.696), grade (0.656), stage (0.656) and other clinical indicators (**Figure 3C**). These results suggest that the risk score of ferroptosis-related genes is an independent predictor for ccRCC patients’ survival.

### Construction of Nomograph

**Figure 4:**
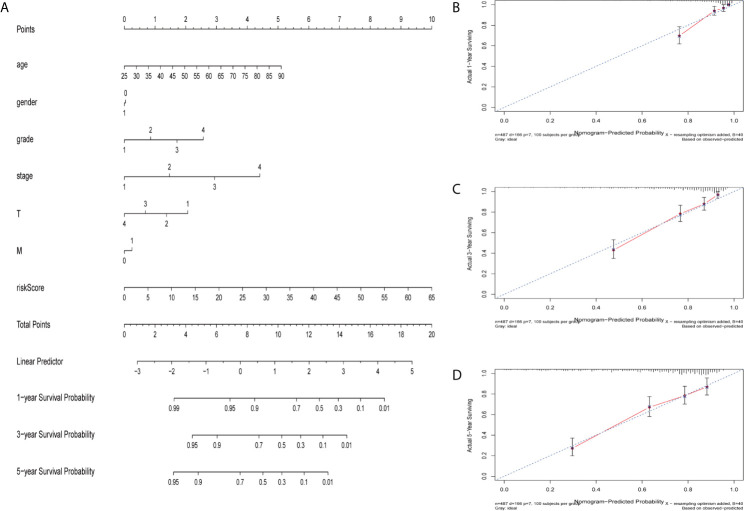
Construction and verification of nomogram. **(A)** The prognostic nomogram constructed based on the risk score of ferroptosis-related genes and clinicopathological parameters predicted the survival rate of ccRCC patients at 1, 3, and 5 years. **(B–D)** Calibration curves showed the concordance between predicted and observed 1, 3 and 5-years survival rates.

The nomogram is a quantitative patient prognosis method (22). In order to accurately estimate the survival probability of patients with ccRCC at 1, 3 and 5 years respectively, we integrated the prognostic characteristics of ferroptosis-related genes and other clinicopathological factors, including age, gender, grade, stage, T and M stages, to construct a nomogram (**Figure 4A**). The concordance index (C-index) value for the nomogram was 0.77. Actual values of the 1-, 3-, and 5-year calibration curve were highly consistent with the predicted values (**Figures 4B–D**), indicating the nomograph we built is reliable and accurate. This may help clinical practitioners make clinical decisions for ccRCC patients and provide valuable insights for patients’ personalized treatment.

### Validation of the Predictive Value and Expression of Ferroptosis-Related Genes

**Figure 5:**
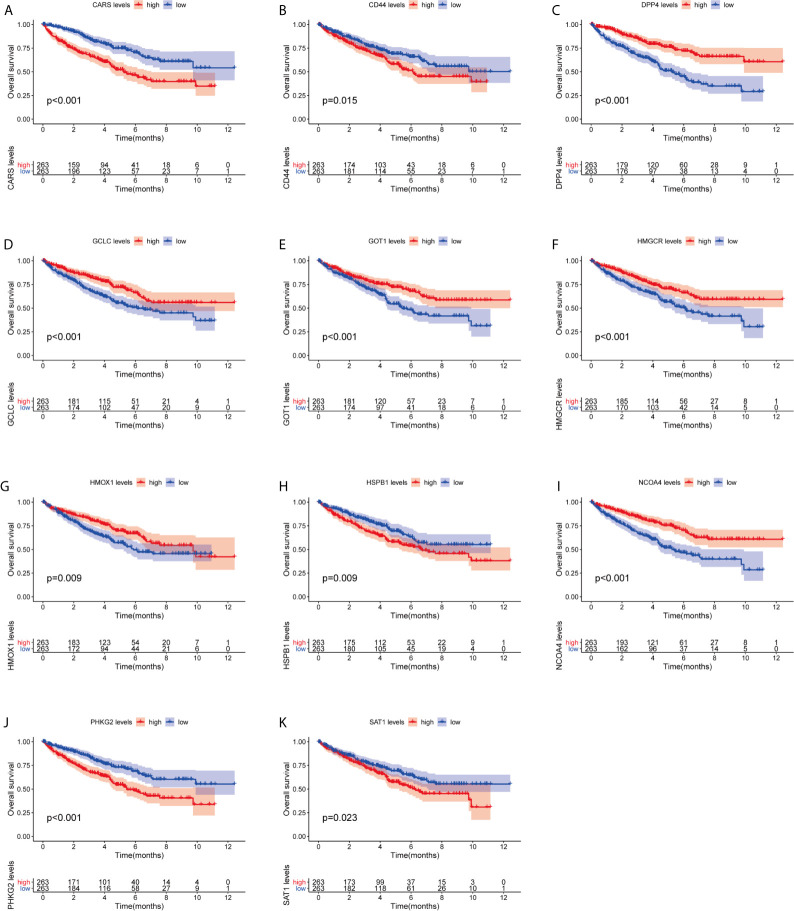
Kaplan–Meier survival curve analysis verified the prognostic value of ferroptosis-related genes in ccRCC. **(A)** CARS, **(B)** CD44, **(C)** DPP4, **(D)** GCLC, **(E)** GPT1, **(F)** HMGCR, **(G)** HMOX1, **(H)** HSPB1, **(I)** NCOA4, **(J)** PHKG2, **(K)** SAT1.

**Figure 6:**
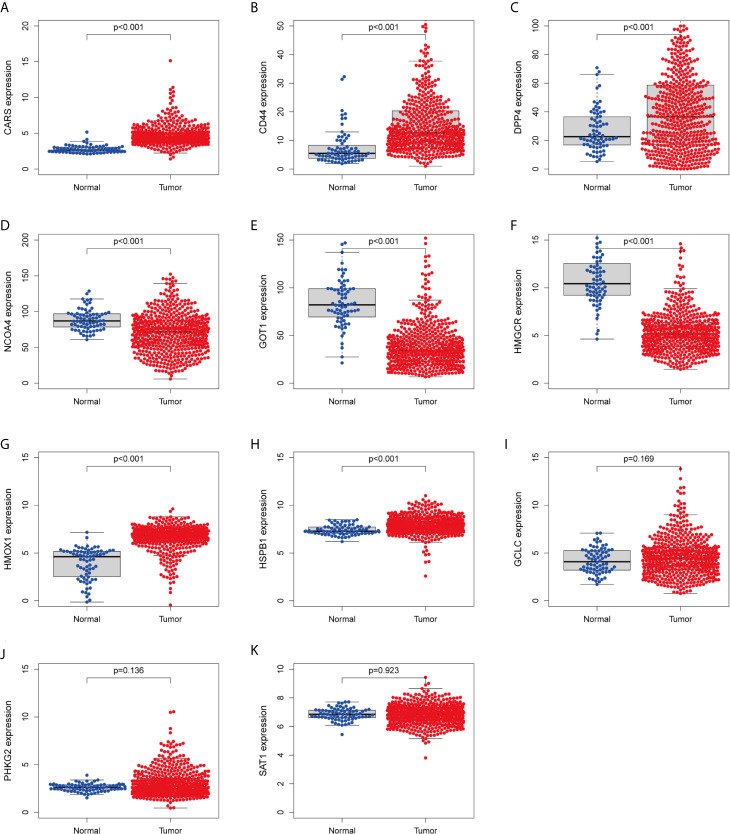
Verify the transcriptional expression of ferroptosis-related in ccRCC and normal kidney tissues. **(A)** CARS, **(B)** CD44, **(C)** DPP4, **(D)** NCOA4, **(E)** GOT1, **(F)** HMGCR, **(G)** HMOX1, **(H)** HSPB1, **(I)** GCLC, **(J)** PHKG2, **(K)** SAT1.

**Figure 7:**
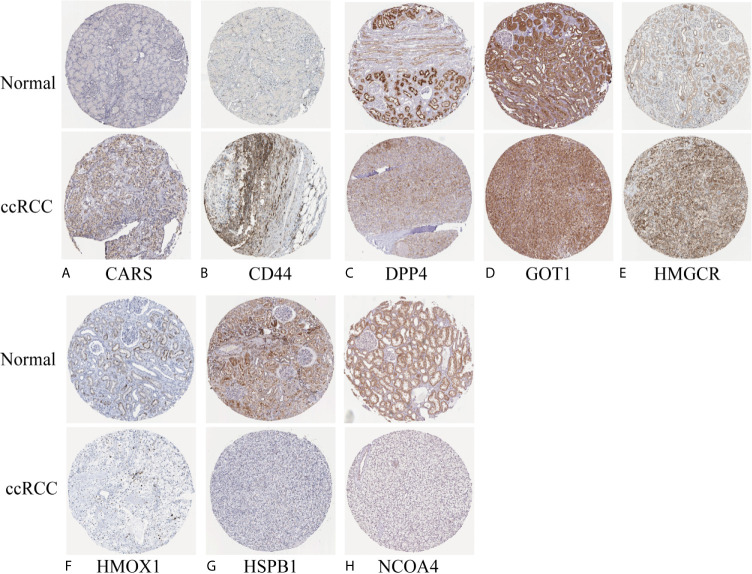
Verify the translational expression of ferroptosis-related in ccRCC and normal kidney tissues. **(A)** CARS, **(B)** CD44, **(C)** DPP4, **(D)** GOT1, **(E)** HMGCR, **(F)** HMOX1, **(G)** HSPB1, **(H)** NCOA4.

We investigated the predictive value of the 11 ferroptosis-related genes in ccRCC. Kaplan–Meier survival curve was used to determine the relationship between gene and OS. As shown in **Figures 5A–K**, all the 11 ferroptosis-related genes were significantly associated with OS in ccRCC patients. Then we further analyzed the expression of these genes between normal tissues and cancer tissues. Beeswarm plot was used for display. Our results showed that eight out of 11 genes (CARS, CD44, DPP4, GOT1, HMGCR, HMOX1, HSPB1, NCOA4) (**Figures 6A–H**) exhibited differences at the transcription level. The former five of these genes were up-regulated in tumors and the latter three were down-regulated. The remaining three genes (GCLC, PHKG2, SAT1) had no difference at the transcription level (**Figures 6I–K**). Finally, we analyzed the expression of these genes at protein level using human protein atlas database. Immunohistochemical results were consistent with the transcriptional levels. Expressions of CRAs, CD44, DPP4, GOT1 and HMGCR were significantly increased in renal clear cell carcinoma compared with those in normal kidney tissue (**Figures 7A–E**). Protein expressions of HMOX1, HSPB1 and NCOA4 were markedly decreased in cancer tissues (**Figures 7F–H**).

### Functional Enrichment Analysis and Gene Set Variation Analysis (GSVA)

**Figure 8:**
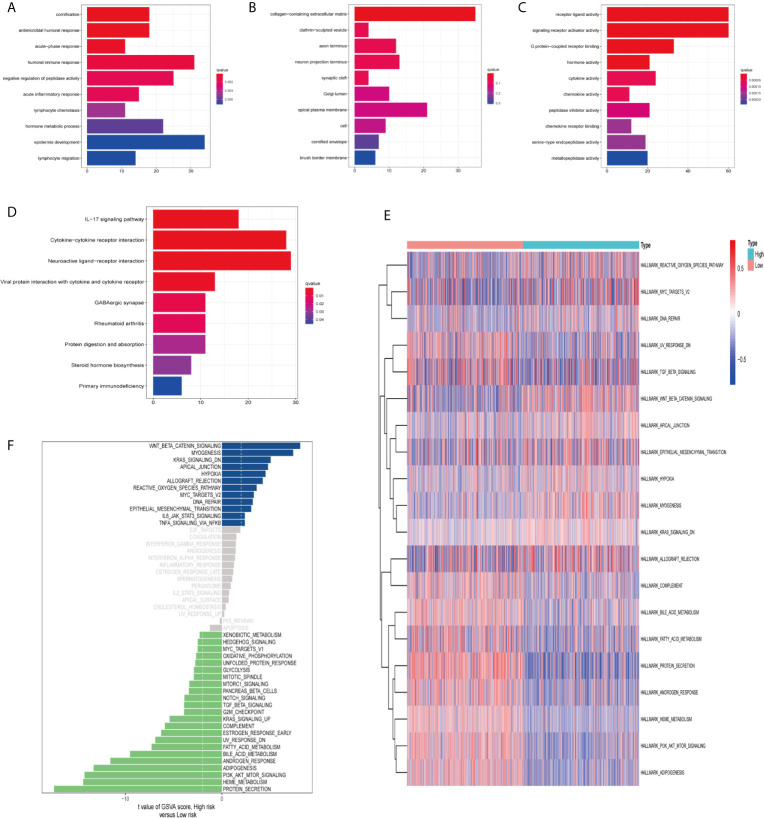
Functional enrichment analysis and Gene Set Variation Analysis (GSVA). Go enrichment analysis of differentially expressed genes, including **(A)** Biological Process (BP), **(B)** Cellular Component (CC), **(C)** Molecular Function (MF). **(D)** KEGG enrichment analysis of differentially expressed genes. GSVA analyzed the relationship between prognostic gene characteristics and tumor characteristics. **(E)** Heat map. **(F)** Histogram.

In order to clarify the biological functions and pathways related to risk scores, DEGs between the high-risk group and the low-risk group were used for GO enrichment and KEGG pathway analysis. DEGs were enriched in the biological processes (BP) with antimicrobial humoral response and humoral immune response. In addition, they were enriched in molecular functions (MF) with many receptor-binding such as G protein-coupled receptor binding and chemokine receptor binding (adjust *P*-value <0.05; **Figures 8A–C** **)**. KEGG pathway analysis also showed that receptor interaction pathways were significantly enriched, such as viral protein interaction with cytochrome and cytochrome receptor, neuroactive ligand–receptor interaction, and cytokine–cytokine receptor interaction (adjust *P*-value <0.05; **Figure 8D**).

Finally, we used GSVA (hallmark gene set) to briefly analyze the relationship between prognostic gene characteristics and cancer characteristics. The heat map showed that there were significant differences in metabolic processes in high and low risk groups, such as bile acid metabolism, fatty acid metabolism, and heme metabolism (**Figure 8E**). The histogram displayed the gene sets enriched in high and low risk groups (**Figure 8F**). We evaluated the correlation between tumor feature score and risk score. Correlation coefficient >0.45 was used as the screening criterion. There was a significant correlation between the six gene sets and the risk score, suggesting that the activation of these features may be involved in the progression of the tumor, and may affect the survival of ccRCC patients (**Figures S3A–F**).

## Discussion

Ferroptosis is a recently defined, non-apoptotic, regulated cell death process that comprises abnormal metabolism of cellular lipid oxides catalyzed by iron ions or iron-containing enzymes (23). In recent years, studies have found that ferroptosis is related to various pathological conditions. The most studied are in the fields of neurodegenerative diseases (24–26) and cancer (27, 28). Therefore, ferroptosis related biomarkers are potential diagnostic biomarkers and therapeutic targets for ccRCC patients. In this study, we systematically analyzed the predictive accuracy of ferroptosis-related genes in ccRCC using bioinformatics and statistical tools.

We utilized the ccRCC patient sample data in TCGA database to obtain the expression levels of ferroptosis-related genes. Based on the lowest AIC value, a COX regression model consisting of 11 genes was established. By calculating the risk score of every ccRCC patient based on these 11 genes expressions in the prognostic signature, the patients were divided into high-risk group and low-risk group according to the median risk score. Kaplan–Meier survival curve showed entirely different survival rates for high- and low-risk patients. Principal Component Analysis (PCA) proved that high and low risk groups were two different components. ROC curve verified the accuracy of the model. Then we analyzed the correlation between prognosis signature and clinicopathological features and found that the prognosis signature we constructed could be widely applicable in different groups with diverse clinical features. Moreover, we combined other clinicopathological features and prognostic signatures for multivariate Cox analysis and proved that the ferroptosis prognostic signature was an independent predictor of survival in ccRCC patients.

Nomogram is a visual display of complex mathematical formulas (29). The reasonable root of using nomogram as diagnostic and prognostic tools is that it effectively promotes the communication between doctors and patients, simplifying the medical process (30, 31), and helping doctors make decisions in diagnosis and treatment (22). Therefore, we established a nomogram consisting of multiple clinical variables such as age, gender, grade, stage, T stage and M stage, and a risk score based on the prognosis signature of ferroptosis-related genes. The calibration curve showed that the actual 1-, 3-, and 5-year survival rates were highly consistent with those predicted rates. This indicates that the prognosis characteristics of ferroptosis-related genes may correctly predict the prognosis of ccRCC patients, and shows great clinical application potential, which provides valuable references for clinical practitioners to make clinical decisions.

In recent years, ferroptosis has shown great potential in tumor therapy, especially in malignant tumors which are resistant to traditional therapies (11). In addition to ferroptosis-inducing factors (10), some other genes have been identified as regulatory molecules for iron deposition. For example, direct targeting of GPx4 may be more effective than interfering with glutathione (32), which reflects the importance of functional researches about ferroptosis-related genes. Wu et al. constructed a survival model using five ferroptosis-related genes (FANCD2, HMGCR, SLC7A11, CARS and NCOA4). Among them, CARS, HMGCR and NCOA4 also existed in our model, indicating the importance of their prognostic features.

In our study, we analyzed the expression of ferroptosis-related genes at both transcription and protein levels. Five genes including CARS, CD44, DPP4, HSPB1, and HMOX1 were up-regulated in ccRCC, while GOT1, HMGCR and NCOA4 were down-regulated. Kaplan–Meier survival curve showed that these 11 ferroptosis-related genes were significantly associated with OS in ccRCC patients, reflecting a huge prognostic value. We also enriched the differentially expressed genes in high and low risk groups, and analyzed the correlation between risk score characteristics and tumor characteristics. The results showed that adiogenesis, androgen response, bill acid metabolism, heme metabolism, and PI3K-Akt-mTOR signaling were significantly correlated with risk score, which suggested that these characteristics might be related to the occurrence of ccRCC.

Our research has some limitations. First, the results would be better to be further verified in other independent datasets. Second, further biochemical experiments can help us to understand the biological function of ferroptosis-related genes.

In conclusion, we constructed a risk model of ferroptosis prognostic signature. In this model, we identified 11 out of 60 ferroptosis-related genes which can accurately predict the survival outcome of ccRCC patients. In addition, the established nomogram can provide personalized suggestions for ccRCC patients’ treatment. Therefore, these 11 ferroptosis-related genes are promising prognostic and diagnostic biomarkers for ccRCC patients.

## Data Availability Statement

The original contributions presented in the study are included in the article/**Supplementary Material**. Further inquiries can be directed to the corresponding author.

## Author Contributions

CY performed the statistical analyses and drafted the manuscript. KC supervised the statistical analyses. KC participated in data analysis and interpretation and provided critical feedback. KC and CY provided data for fine-mapping. All authors contributed to the article and approved the submitted version.

## Conflict of Interest

The authors declare that the research was conducted in the absence of any commercial or financial relationships that could be construed as a potential conflict of interest.
