# Supplementary figures and images for: Ferroptosis-Related Gene Signature Accurately Predicts Survival Outcomes in Patients With Clear-Cell Renal Cell Carcinoma
Source: Front Oncol. 2021 Apr 30;11:649347. doi: 10.3389/fonc.2021.649347 (PMC8120155; doi:10.3389/fonc.2021.649347)

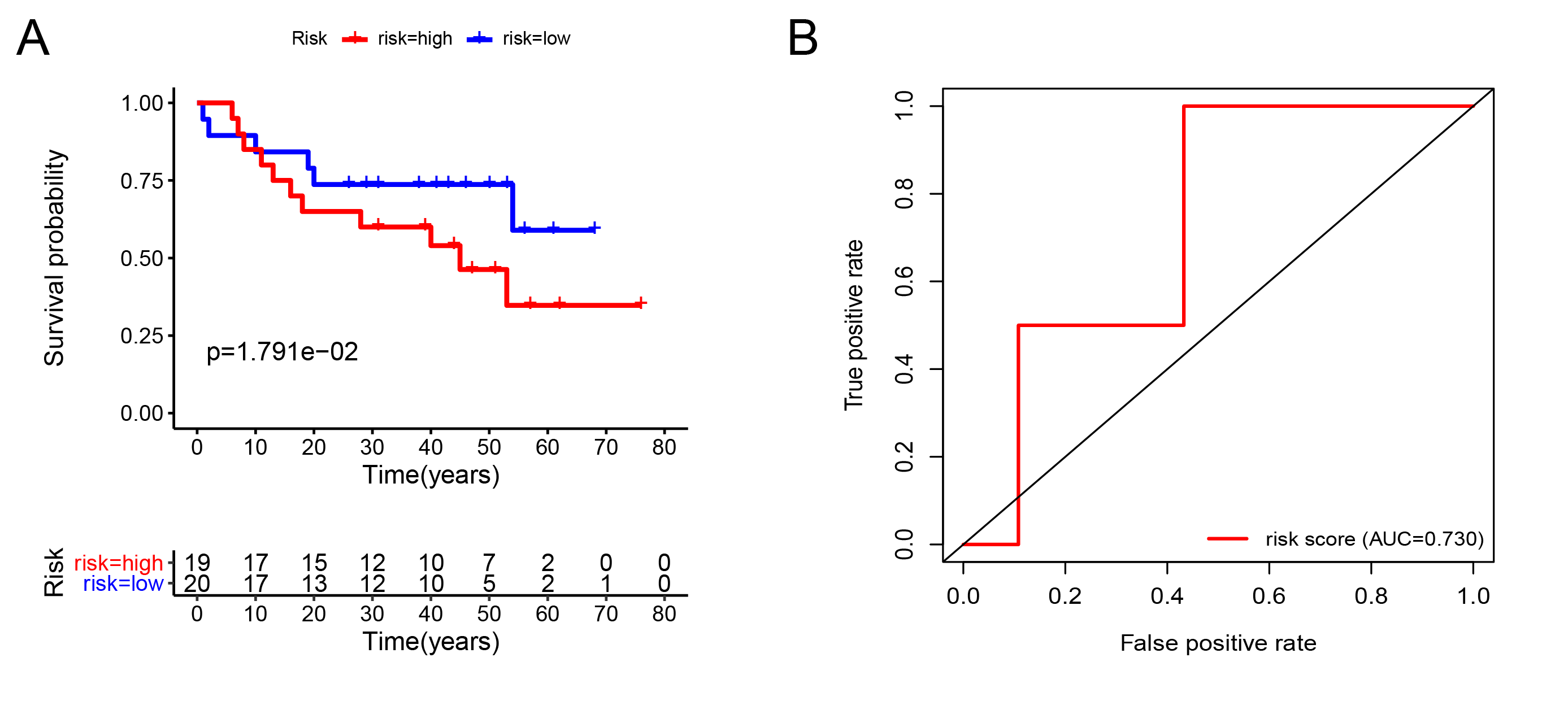

Supplement: Supplementary file 1 [file Image_1.tif]

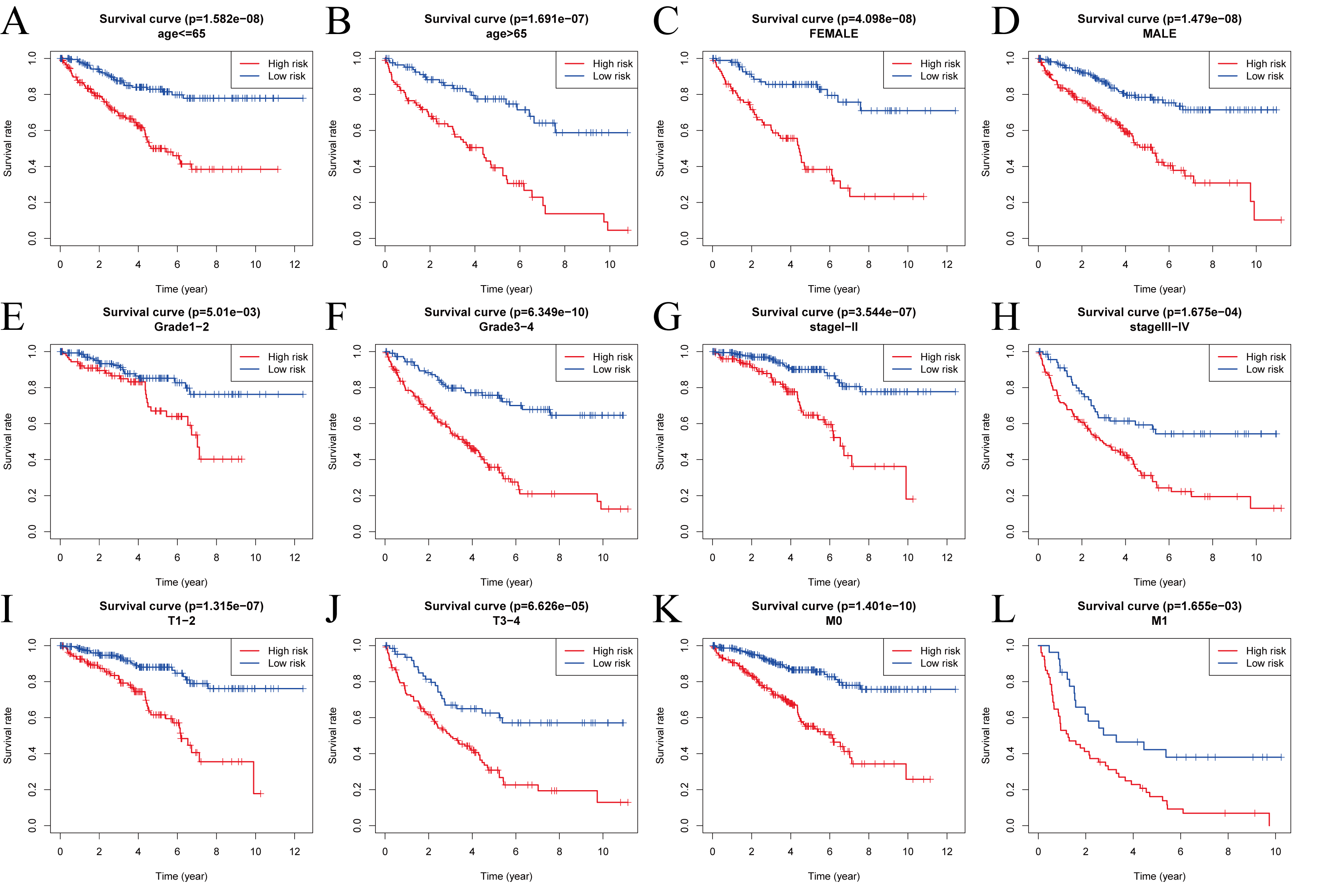

Supplement: Supplementary file 2 [file Image_2.tif]

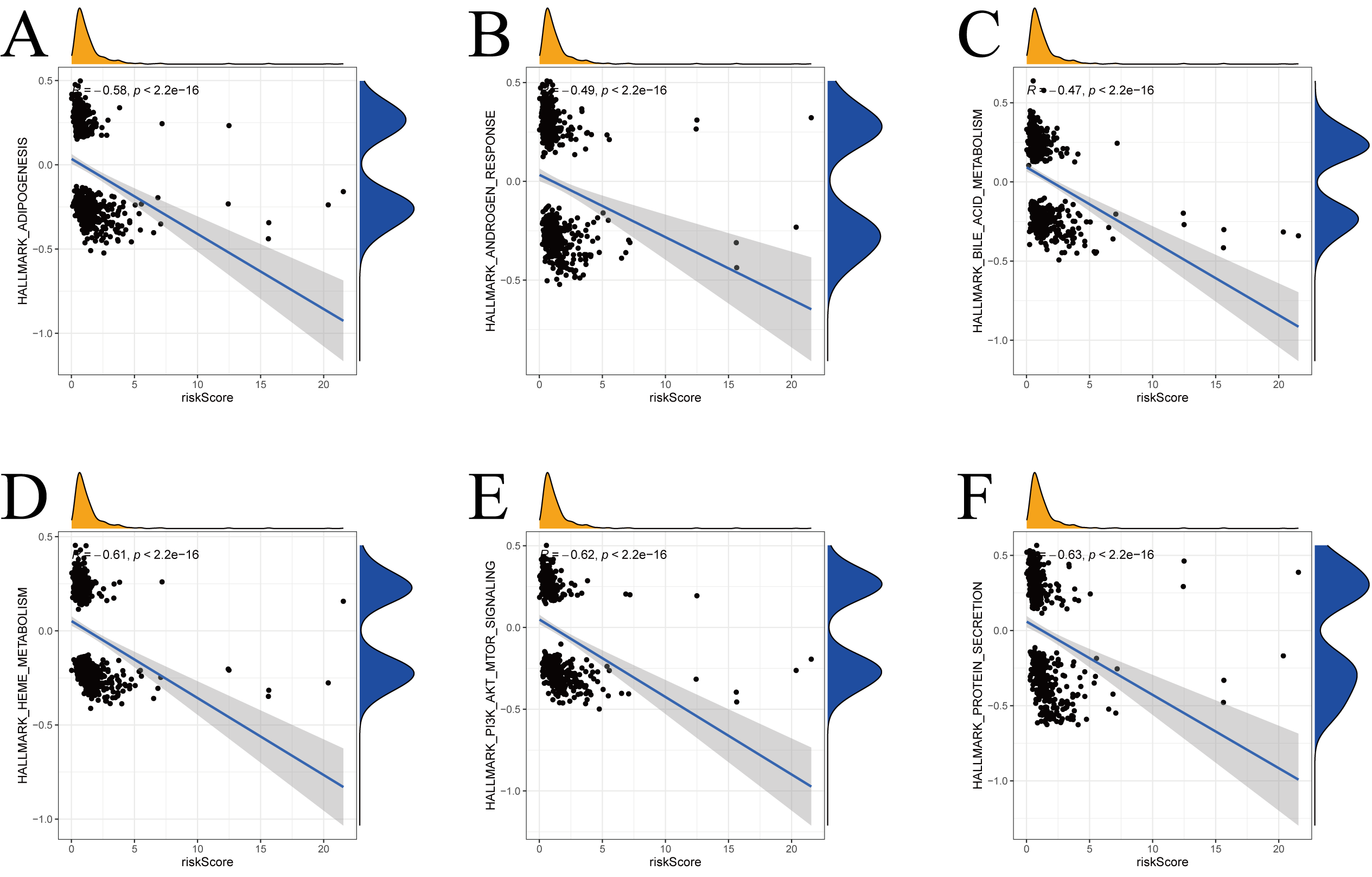

Supplement: Supplementary file 3 [file Image_3.tif]
